# Additional corpus biopsy enhances the detection of *Helicobacter pylori* infection in a background of gastritis with atrophy

**DOI:** 10.1186/1471-230X-12-182

**Published:** 2012-12-29

**Authors:** Hung-Chieh Lan, Tseng-Shing Chen, Anna Fen-Yau Li, Full-Young Chang, Han-Chieh Lin

**Affiliations:** 1 Division of Gastroenterology, Department of Medicine, Taipei Veterans General Hospital and National Yang-Ming University, #201 Shih-Pai Road, Section 2, Taipei, Taiwan, ROC; 2 Department of Pathology and Laboratory Medicine, Taipei Veterans General Hospital and National Yang-Ming University, Taipei, Taiwan, ROC; 3 Division of General Medicine, Department of Medicine, Taipei City Hospital, Taipei, Taiwan, ROC

**Keywords:** Gastritis with atrophy, Biopsy-based test, Biopsy site, *Helicobacter pylori*

## Abstract

**Background:**

The best sites for biopsy-based tests to evaluate *H. pylori* infection in gastritis with atrophy are not well known. This study aimed to evaluate the site and sensitivity of biopsy-based tests in terms of degree of gastritis with atrophy.

**Methods:**

One hundred and sixty-four (164) uninvestigated dyspepsia patients were enrolled. Biopsy-based tests (i.e., culture, histology Giemsa stain and rapid urease test) and non-invasive tests (anti-*H. pylori* IgG) were performed. The gold standard of *H. pylori* infection was defined according to previous criteria. The sensitivity, specificity, positive predictive rate and negative predictive rate of biopsy-based tests at the gastric antrum and body were calculated in terms of degree of gastritis with atrophy.

**Results:**

The prevalence rate of *H. pylori* infection in the 164 patients was 63.4%. Gastritis with atrophy was significantly higher at the antrum than at the body (76% vs. 31%; *p*<0.001). The sensitivity of biopsy-based test decreased when the degree of gastritis with atrophy increased regardless of biopsy site (for normal, mild, moderate, and severe gastritis with atrophy, the sensitivity of histology Giemsa stain was 100%, 100%, 88%, and 66%, respectively, and 100%, 97%, 91%, and 66%, respectively, for rapid urease test). In moderate to severe antrum or body gastritis with atrophy, additional corpus biopsy resulted in increased sensitivity to 16.67% compare to single antrum biopsy.

**Conclusions:**

In moderate to severe gastritis with atrophy, biopsy-based test should include the corpus for avoiding false negative results.

## Background

*Helicobacter pylori (H. pylori)* and gastritis with atrophy are both risk factors for gastric cancer [1]. Around one-third of infected patients are estimated to have gastritis with atrophy [2] and the regression of these pre-neoplasm lesions may occur after successful eradication [3-5]. As such, *H. pylori* eradication in patients with gastritis with atrophy is recommended by current guidelines [1,6,7].

Although eradication is essential for this group of patients, the accurate identification of *H. pylori* against a background of gastritis with atrophy remains difficult [8]. False negative results occur even under more reliable *H. pylori* diagnostic tests like histology Giemsa stain [9]. False negative status, or the so-called sampling error, may result from patchy bacterial colonization through the stomach and altered distribution because of gastritis with atrophy and intestinal metaplasia. Thus, it is important to set a recommended biopsy site.

The updated Sydney Classification had set the gold standard for gastric biopsy more than 10 years ago [10]. According to this Classification, five biopsy sites should be collected: one specimen each should be obtained from the lesser and the greater curvature of the antrum, both within 2–3 cm form the pylorus; from the lesser curvature of the corpus about 4 cm proximal to the angulus; from the middle portion of the greater curvature of the corpus, approximately 8 cm from the cardia; and one from incisura angularis. However, for the consideration of patient’s comfort and operator’s convenience, this kind of extensive approach is uncommon in our daily practice. Studies on the most practical biopsy site for diagnosing *H. pylori* infection have conflicting results. Antrum biopsy is recommended by Genta et al. [11] while others recommend at least one corpus biopsy [12,13]. Hazell et al. and Woo et al. found it necessary to take both antral and corpus biopsies [14,15]. According to current guidelines, there is no optimal site when performing a biopsy-based test in a general condition, much less in those with gastritis with atrophy [16].

It is suggested that as gastritis with atrophy progresses, the mid corpus is the last area involved and is the last “lodgeable” mucosa for *H. pylori* in the stomach [17,18]. Additional corpus biopsy is suggested in these situations [12] but the exact additional benefit is not well known.

This prospectively designed study used the combination method as gold standard to investigate the correlation among sensitivity of biopsy-based test, biopsy location, degree of gastritis with atrophy, and *H. pylori* prevalence rate.

## Methods

### Patient population

Dyspeptic patients scheduled for upper gastrointestinal endoscopy were recruited. Patients with any of the following conditions were excluded: (1) ulcer complications (e.g., bleeding, stenosis, or perforation); (2) previous stomach surgery; (3) gastric neoplasms; (4) use of any substituted benzimidazoles and bismuth-containing preparations within the last 7 days prior to the start of the study; (5) past or current treatment with anti-*H. pylori* therapy; or (6) severe systemic diseases. All patients provided prior informed consent and received invasive and non-invasive tests for *H. pylori*. The local Ethics Committee on Human Test approved the study.

### Endoscopy and biopsy sampling

According to the updated Sydney system, the biopsy sampling protocol required that more than five biopsy samples were obtained: two from the antral mucosa; one from the mucosa of the angularis incisura; two from the oxyntic area. For the consideration of patients’ comfort and operator’s convenience, we modified this recommendation. Two biopsy sites with multiple biopsies were applied to our endoscopy biopsy protocol. Three sets of biopsy specimens each from the greater curvature of the mid-body and lesser curvature of the antrum near the incisura were obtained during endoscopy for urease, histologic, and culture tests.

### Rapid urease test

One biopsy from the greater curvature of the mid-body and one from the lesser curvature near the incisura were obtained for the urease test (CLO test; Delta West, Bentley, Australia). Antral and body biopsy specimens were evaluated separately, and the test was considered positive when the color changed from orange to pink within 24 hours.

### Histologic evaluation of the biopsy samples

Biopsy specimens from the antrum and the body were fixed in formalin and assessed for the presence of *H. pylori* by a modified Giemsa stain, and for the degree of inflammatory cell infiltration, atrophy, and intestinal metaplasia by hematoxylin and eosin staining. The antrum and body histologic features of gastric mucosa were graded according to the updated Sydney System (0, none; 1, mild; 2, moderate; and 3, severe) [10]. In addition, the degree of gastritis activity was evaluated in Giemsa-negative patients with positive for either rapid urease test or serology. Also, the activity of gastritis was recorded according to the updated Sydney System. (1–4 points with 1 representing “normal”, 2 to 4 representing “mildly, moderately and markedly active gastritis”, respectively). Those who got two or more points on the pathological review were regarded as active gastritis which is an indirect sign of *H. pylori* infection. An experienced pathologist (Anna Fen-Yau Li) who was blinded to the results of other tests for *H. pylori* evaluated all histologic sections.

### Culture

In culture, the biopsy sample was homogenized with 0.3 mL broth, plated on chocolate agar, and incubated at 37°C in a micro-aerobic (15% CO_2_ and 5% O_2_) incubator until the colony appeared, which was usually 3 days. The negative plates were kept for 7 days. The growth of *H. pylori* was confirmed by the characteristic morphology (Gram-negative and curved) and positive catalase, oxidase, and urease reactions observed.

### Serologic evaluation

Serum specimens were tested for the presence of IgG antibodies against *H. pylori* using a quantitative ELISA test (HEL-pTEST II; AMRAD, Kew, Australia) according to the manufacturer’s instructions. A specimen was considered positive if it contained >50 units/mL and negative if it contained ≤50 units/mL.

### Serum pepsinogen levels

For serology studies, blood was drawn immediately after endoscopy and collected and stored at −70°C until assay. Fasting serum Pepsinogen I was measured in all patients by radioimmunoassay (Pepsik; Sorin Biomedica, Saluggia, Italy) according to the manufacturer’s instructions, while basal Pepsinogen II levels were determined using a specific enzyme immunoassay (BIOHIT Plc, Helsinki, Finland).

### Gold standard definition

To define the gold standard of active *H. pylori* infection, we modified the approach used by Shin et al. before [8]. A patient was classified as current *H. pylori* infection based on either a positive culture or, in the case of a negative culture, both positive histology and positive urease test (Group A). A patient was classified as *H. pylori*-negative when the culture, histologic examination, urease test, and serology were all negative (Group E). When either the histologic examination (Giemsa stain or presence of active gastritis) or urease test was positive, the results were classified based on the results of serology test. When IgG antibodies to *H. pylori* were detected, the results were considered “probably positive” (Group B). When IgG antibodies were not detected, the results were regarded as being “probably negative” (Group C). When a patient was serologically positive but negative on all biopsy-based tests, this was interpreted as either a past *H. pylori* infection or a false-positive result and the patient was classified as negative for current *H. pylori* infection (Group D). Groups A and B were defined as gold standard positive while Groups C, D, and E were gold standard negative.

### Statistical analysis

Standard methods were used to calculate the sensitivity, specificity, and positive and negative predictive values, their 95% confidence intervals, and their test validity. Chi-square tests were used to compare variables (i.e., number of cases who had moderate-to-marked gastritis with atrophy at the antrum and corpus). To examine the decreasing trend in the prevalence of histology, as influenced by the severity of glandular atrophy, chi-square test for trend was applied. All statistical analyses and database collection were performed using the Statistical Package for Social Sciences (SPSS 17.0 for Windows, SPSS. Inc., Chicago, IL, USA).

## Results

### Subjects characteristics

**Figure 1:**
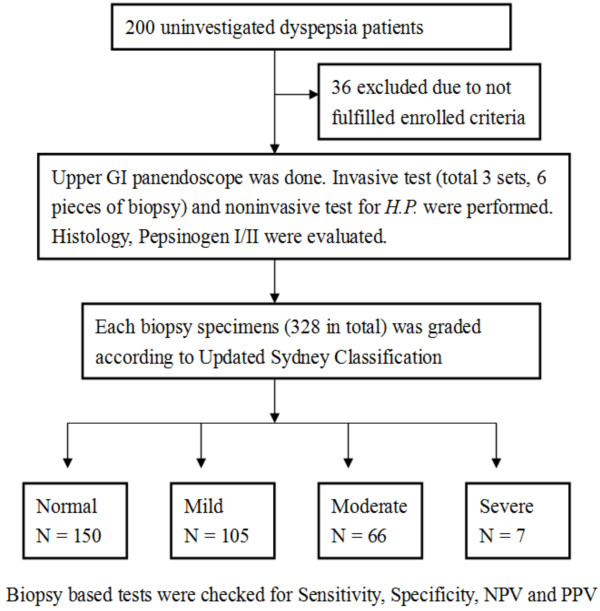
**Flow chart of the enrolled patients and the grouping of biopsy specimens according to the degree of gastritis with atrophy.***H.P.*, *Helicobacter pylori*; NPV, negative predictive rate; PPV, positive predictive rate.

A total of 164 patients were enrolled in this study. Regardless of the biopsy site, a total of 328 biopsy specimens were received for histologic evaluation (Figure 1).

**Figure 2:**
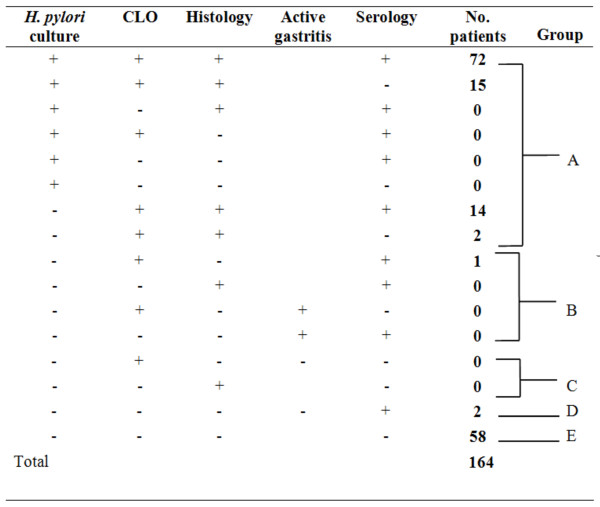
**Diagnostic tests for Helicobacter pylori detection:** Group A, definitely positive (n=103); Group B, probably positive (n=1); Group C, probably negative (n=0); Group D, past *H. pylori* infection or false positive (n=2); Group E, definitely negative (n=58); CLO, indicates rapid urease test on biopsy specimens; Histology, refers to *H. pylori* positive based on modified Giemsa staining; Active gastritis, refers to presence or absence of active gastritis (an indirect sign of active *H. pylori* infection).

Using the pre-defined gold standard, the results of the four diagnostic tests for detecting current *H. pylori* infection in all subjects (n=164) were shown in Figure 2. Culture was positive in 87 patients. Among the patients who were culture negative, 16 were positive for both the CLOtest and histology. These 103 patients were regarded as true positives (Group A). Moreover, one patient was positive on serology and positive on either the CLOtest or histology testing (Giemsa stain or presence of active gastritis), so this patient was classified as probably *H. pylori* positive (Group B). No patient was positive for a single biopsy-based test, so there was no probably negative patient (Group C). Two patients were positive on serology only (with titers of 67 unit/ml and 171 unit/ml, respectively). Their gastritis activity of the biopsy specimens were reviewed again. None of them showed active gastritis in the pathological review (both of them got 1 point at their antrum and body biopsy specimens). They were considered either a past *H. pylori* infection or a false positive (Group D). Fifty-eight patients were negative on all tests and were considered true negative (Group E).

**Figure 3:**
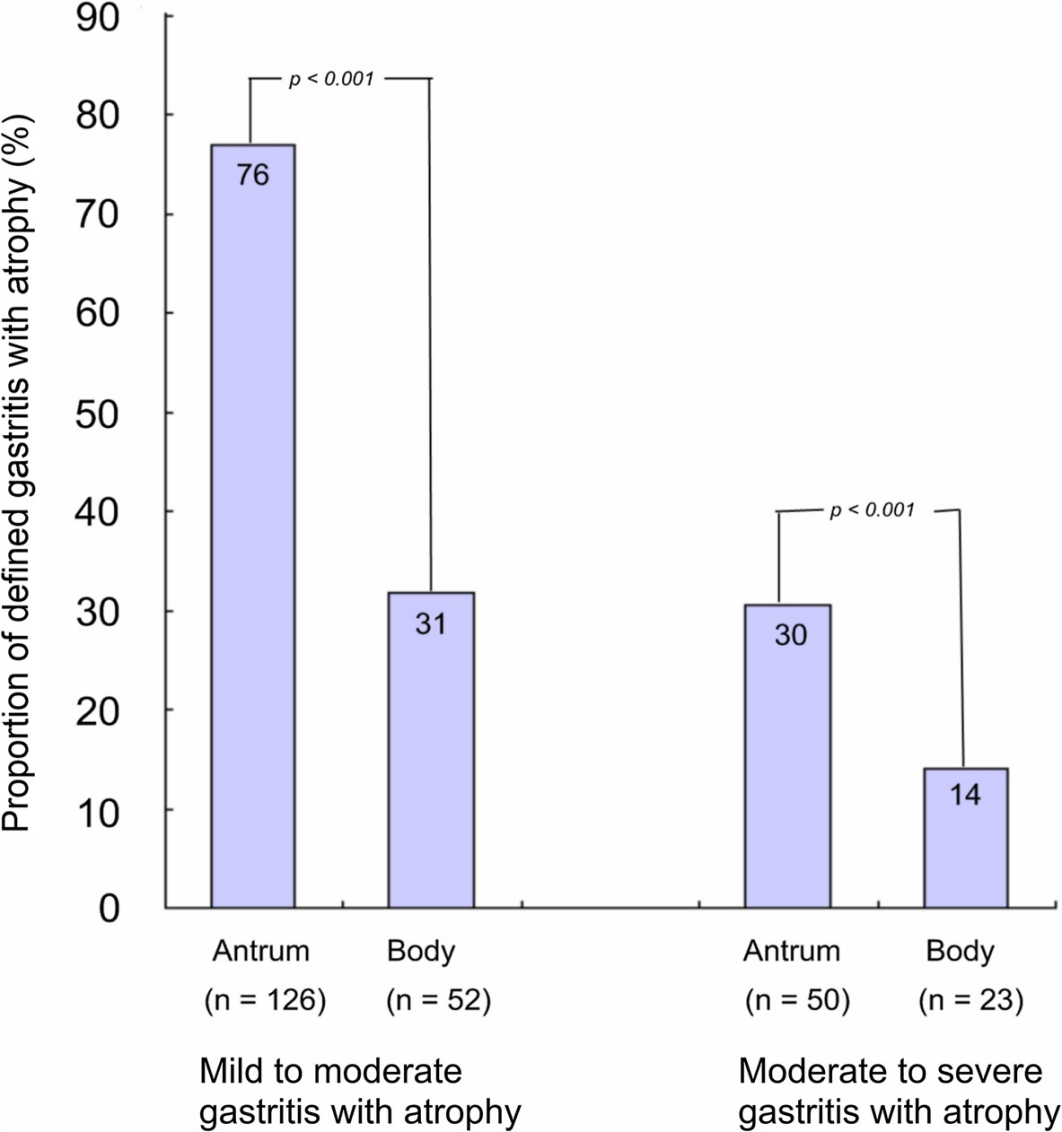
**The proportion of mild-severe and moderate-severe gastritis with atrophy based on biopsy site.** The *p* value was calculated using the chi-square test.

When Groups A and B were considered as *H. pylori* positive and groups C, D, and E as *H. pylori* negative, the patients’ characteristics, including numbers of *H. pylori* infection, mean ratio of Pepsinogen I/II, and numbers of peptic ulcer and gastritis with atrophy were shown in Table 1. The overall *H. pylori* infection rate was 63.4% and the mean Pepsinogen I/II ratio was 2.37. According to Updated Sydney Classification, 50 patients (31.5%) had moderate-to-severe antrum gastritis with atrophy, and 23 (14.0%) had moderate-to-severe body gastritis with atrophy. Compared to the body, the antrum had significantly higher percentage of gastritis with atrophy (Figure 3). The antrum also had significantly higher percentage of intestinal metaplasia (antrum 11.6% vs. body 0%, *p*<0.001).

**Table 1:** Demographic characteristics of patients (n=164)

**Age-year, mean (range)**	**43.51**	**(20–70)**
Gender-no. (%)		
Male	85	(51.8)
Female	79	(48.2)
PgI/PgII ratio (range)		
-Mean	2.37	(0.46–11.51)
*H. pylori* status		
Infection	104	(63.4)
Non-infection	60	(36.6)
Total histology		
specimens and *H. pylori* status		
Infection	208	(63.4)
Non infection	120	(36.6)
Peptic ulcer, no. (%)		
GU	7	(4.2)
DU	53	(32.3)
Antrum gastritis with atrophy		
Normal to mild	114	(69.5)
Moderate to severe	50	(31.5)
Body gastritis with atrophy		
Normal to mild	141	(86.0)
Moderate to severe	23	(14.0)
Antrum intestinal metaplasia		
Normal	145	(88.4)
Intestinal metaplasia	19	(11.6)
Body intestinal metaplasia		
Normal	164	(100.0)
Intestinal metaplasia	0	(0)

### Sensitivity of biopsy based tests and degree of gastritis of atrophy

**Figure 4:**
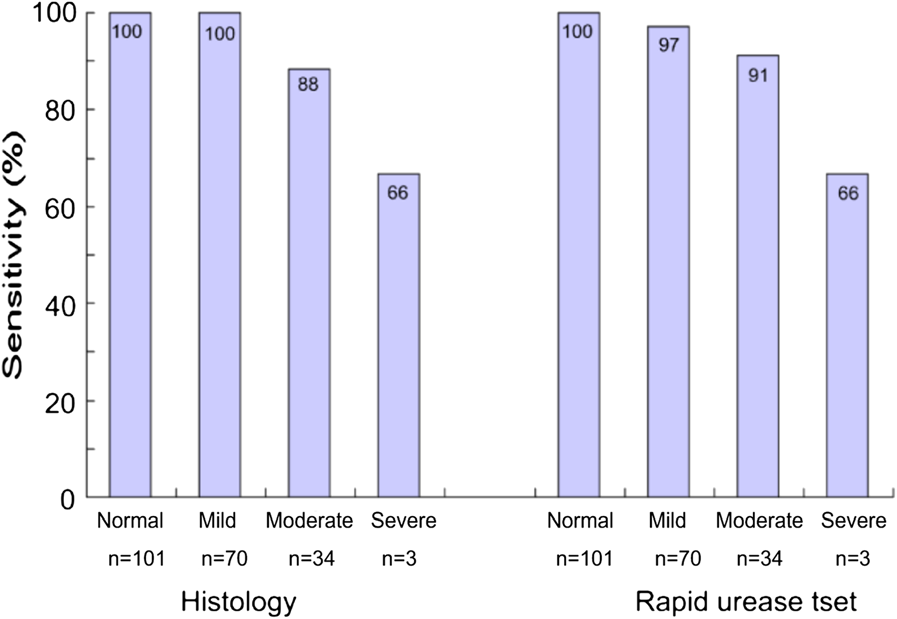
The sensitivity of biopsy-based tests (histology Giemsa stain and rapid urease test) according to grade of mucosal atrophy.

Regardless of biopsy site, the biopsy specimens (n=328) were grouped according to the degree of gastritis with atrophy. Of these, 208 specimens were collected from the “defined infection” patients (Groups A and B). In these biopsy specimens, 101 had normal histology while 70 had mild, 34 had moderate, and 3 had severe gastritis with atrophy. The sensitivity of histology Giemsa stain and rapid urease test in these specimens were evaluated (Figure 4) As the degree of gastritis with atrophy progressed, the sensitivities of these two biopsy-based test decreased (sensitivity of histology Giemsa stain in normal, mild, moderate, and severe gastritis with atrophy was 100%, 100%, 88%, and 66%, respectively, while the sensitivity of rapid urease test was 100%, 97%, 91%, and 66%, respectively).

### Sensitivity of biopsy based tests and biopsy sites

Using the pre-defined gold standard, the sensitivities and specificities of the diagnostic tests, depending on grade of antrum and body atrophy, were presented in Tables 2 and 3. In moderate to severe antrum gastritis with atrophy (n=50), single antrum biopsy for CLOtest yielded fair sensitivity (84%; 95% CI: 63.08-94.75%), which was lower than single body biopsy or combination of body and antrum (100%; 95% CI: 83.42-100%). There was a similar finding in histology (sensitivity of single antrum biopsy, 84% vs. 96% in single body biopsy or combination of body and antrum). However, when the degree of antrum gastritis with atrophy was normal-to-mild, the sensitivity of both biopsy-based tests, regardless of biopsy site, was between 97.47% and 100% (Table 2).

**Table 2:** ***H. pylori *sensitivity and specificity depending on the grade of antrum gastritis with atrophy**

**Ca degree of gastritis with atrophy at antrum**	**Normal to mild**	**Moderate to severe**
**n=164**	**n=114**	**n=50**
**CLOtest Antrum**		
Sensitivity	97.47 (90.31-99.56)	84.00 (63.08-94.75)
Specificity	100.00 (87.68-100.00)	100.00 (83.42-100.00)
PPV	100.00 (94.08-100.00)	100.00 (80.76-100.00)
NPV	94.59 (80.47-99.06)	86.20 (67.43-95.49)
Accuracy	98.25	
**CLOtest Body**		
Sensitivity	100.00 (94.22-100.00)	100.00 (83.42-100.00)
Specificity	100.00 (87.68-100.00)	100.00 (83.42-100.00)
PPV	100.00 (94.22-100.00)	100.00 (83.42-100.00)
NPV	100.00 (87.68-100.00)	100.00 (83.42-100.00)
Accuracy	100.00	100.00
**CLOtest Antrum + Body**		
Sensitivity	100.00 (94.22-100.00)	100.00 (83.42-100.00)
Specificity	100.00 (87.68-100.00)	100.00 (83.42-100.00)
PPV	100.00 (94.22-100.00)	100.00 (83.42-100.00)
NPV	100.00 (87.68-100.00)	100.00 (83.42-100.00)
Accuracy	100.00	100.00
**Histology Antrum**		
Sensitivity	98.73 (92.18-99.93)	84.00 (63.08-94.75)
Specificity	100.00 (87.68-100.00)	100.00 (83.42-100.00)
PPV	100.00 (94.15-100.00)	100.00 (80.76-100.00)
NPV	97.22 (83.80-99.85)	86.20 (67.43-95.49)
Accuracy	99.12	92.00
**Histology Body**		
Sensitivity	100.00 (94.22-100.00)	96.00 (77.68-99.79)
Specificity	100.00 (87.68-100.00)	100.00 (83.42-100.00)
PPV	100.00 (94.22-100.00)	100.00 (82.83-100.00)
NPV	100.00 (87.68-100.00)	96.15 (78.42-99.80)
Accuracy	100.00	98.00
**Histology Antrum + Body**		
Sensitivity	100.00 (94.22-100.00)	96.00 (77.68-99.79)
Specificity	100.00 (87.68-100.00)	100.00 (83.42-100.00)
PPV	100.00 (94.22-100.00)	100.00 (82.83-100.00)
NPV	100.00 (87.68-100.00)	96.15 (78.42-99.80)
Accuracy	100.00	98.00

**Table 3:** ***H. pylori *sensitivity and specificity depending on grade of body gastritis with atrophy**

**Ca degree of gastritis with atrophy at body**	**Normal to mild**	**Moderate to severe**
**n=164**	**n=141**	**n=23**
**CLOtest Antrum**		
Sensitivity	95.66 (88.62-98.60)	83.33 (50.88-97.06)
Specificity	100.00 (90.94-100.00)	100.00 (67.86-100.00)
PPV	100.00 (94.79-100.00)	100.00 (65.55-100.00)
NPV	92.45 (80.93-97.55)	84.61 (53.66-97.29)
Accuracy	97.16	91.30
**CLOtest Body**		
Sensitivity	100.00 (95.00-100.00)	100.00 (69.87-100.00)
Specificity	100.00 (90.94-100.00)	100.00 (67.85-100.00)
PPV	100.00 (95.00-100.00)	100.00 (69.87-100.00)
NPV	100.00 (90.94-100.00)	100.00 (67.85-100.00)
Accuracy	100.00	100.00
**CLOtest Antrum + Body**		
Sensitivity	100.00 (95.00-100.00)	100.00 (69.87-100.00)
Specificity	100.00 (90.94-100.00)	100.00 (67.85-100.00)
PPV	100.00 (95.00-100.00)	100.00 (69.87-100.00)
NPV	100.00 (90.94-100.00)	100.00 (67.85-100.00)
Accuracy	100.00	100.00
**Histology Antrum**		
Sensitivity	96.74 (90.09-99.15)	83.33 (50.88-97.06)
Specificity	100.00 (90.94-100.00)	100.00 (67.86-100.00)
PPV	100.00 (94.84-100.00)	100.00 (65.55-100.00)
NPV	94.23 (83.08-98.50)	84.61 (53.66-97.29)
Accuracy	97.87	91.30
**Histology Body**		
Sensitivity	100.00 (95.00-100.00)	91.67 (59.75-99.56)
Specificity	100.00 (90.94-100.00)	100.00 (67.86-100.00)
PPV	100.00 (95.00-100.00)	100.00 (67.86-100.00)
NPV	100.00 (90.94-100.00)	91.67 (59.75-99.56)
Accuracy	100.00	95.65
**Histology Antrum + Body**		
Sensitivity	100.00 (95.00-100.00)	91.67 (59.75-99.56)
Specificity	100.00 (90.94-100.00)	100.00 (67.86-100.00)
PPV	100.00 (95.00-100.00)	100.00 (67.86-100.00)
NPV	100.00 (90.94-100.00)	91.67 (59.75-99.56)
Accuracy	100.00	95.65

In moderate-to-severe body gastritis with atrophy (n=23), single antrum biopsy for CLOtest yielded fair sensitivity (83.33%; 95% CI: 50.88-97.06%), which was lower than single body biopsy or combination of body and antrum (100%; 95% CI: 69.87-100%). A similar finding was found in histology Giemsa stain (sensitivity of single antrum biopsy, 83.33% vs. 91.67% in single body biopsy or combination of body and antrum). However, when the degree of body gastritis with atrophy was normal to mild (n=141), the sensitivity of both biopsy based tests, regardless of biopsy site, was between 95.66% and 100% (Table 3).

**Figure 5:**
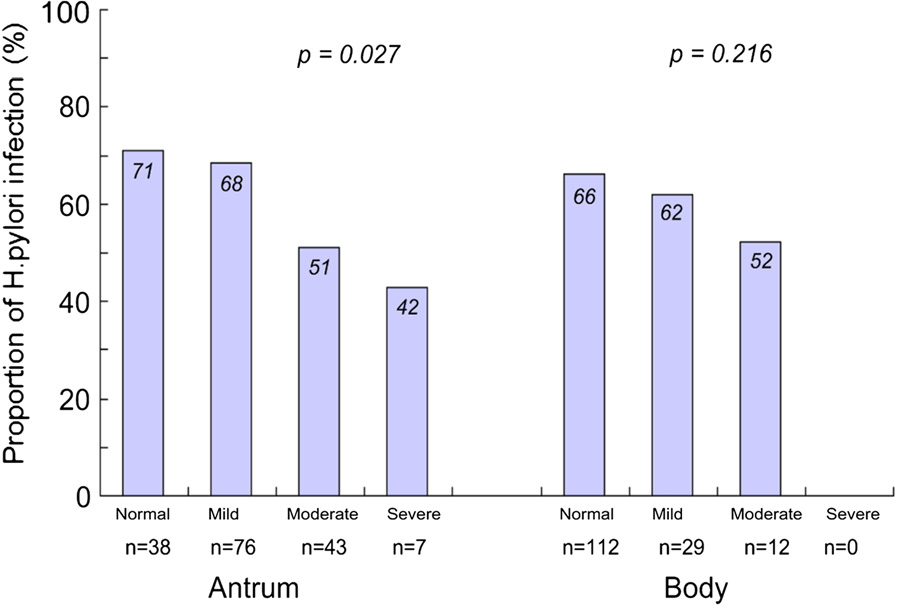
**The prevalence rate of *H. pylori *infection according to grade of antrum and body gastritis with atrophy.** The *p* value was calculated using the chi-square test for trend.

The prevalence rate of *H. pylor*i infection between normal, mild, moderate, and severe gastritis with atrophy were evaluated. As the degree of gastritis with atrophy increased, the prevalence rate of *H. pylori* infection decreased (Figure 5). As the degree of antrum gastritis with atrophy increased, the prevalence rate of *H. pylori* infection decreased significantly (*p=*0.027; chi-square test for trends). A similar trend was found in body gastritis with atrophy, with a trend of decreasing *H. pylori* infection rate (*p=*0.216).

## Discussion

Biopsy-based tests are important diagnostic tools for *H. pylori*. However, the optimal biopsy site is still unknown, especially in cases of gastritis with atrophy. Current guidelines only recommend combining antrum and body biopsies for rapid urease test in the circumstance of antibiotics or proton pump inhibitors exposure. Sampling from the angularis, corpus, and antrum for histology was ever suggested in the same situation [16]. Reviewing recent studies, there is little practical information about the adequate biopsy numbers and suitable biopsy sites. Single and multiple specimens at the antrum alone, at the body alone, or in combination of antrum and body had been described and applied (Table 4). These diagnostic approaches seem largely arbitrarily [8,16,19-28]. In order to clarify the exact benefit of these biopsy sites, a gold standard is necessary.

**Table 4:** **Studies on the diagnostic tests of *H. pylori *and the respective biopsy sites chosen, published between November 2005 and February 2011**

**Authors**	**Country and population studied**	**Sampling site of Histology stain**	**Sampling site of Rapid urease test**	**Age range of subjects (years)**	**Numbers of subjects**	**Numbers of *H. pylori*-positive %**
Choi et al. [22]	Korea, health check-up examinees	*1LA1LB	1LA	Mean 47.8	515	53.2
Hsu et al. [23]	Taiwan, dyspepsia	-	1LA vs. 1LA1GB	Mean 55.0-57.8	355	33.5
Vaira et al. [24]	Italy, dyspepsia	2A	2A	Median 52	1000	45.3
Goh et al. [25]	Malaysia, dyspepsia	1A1B	1A1B	Mean 50.7	206	53.9
Shin et al. [8]	Korea, gastritis with atrophy	2A2B	1A1B	Mean 57.7	651	41.2
Siddique et al. [26]	Kuwait, endoscopy evaluation	-	1A vs. 2A vs. 3A vs. 4A	Mean 36.1	100	-
Yoo et al. [21]	Korea, dyspepsia	2A2B	1LA1LB	Mean 56.8	430	-
Yakoob et al. [27]	Pakistan, dyspepsia	2A	2A	Mean 43	109	57.0
Kim et al. [28]	Korea, gastric cancer	2LA2LB2GB	1GB	Median 61	194	84.0
Tang et al. [29]	Taiwan, bleeding	2B2U	2A	Mean 63.5	324	53.7
Roma-Giannikou et al. [30]	Greece	1A	1A	Mean 10.4	254	-
Chey et al. [15]	Guideline	1GA1GB1Ag	1AgB1GA	-	-	-

*1A, one piece from the antrum; 1B, one piece from the body; 2A2B, two pieces from the antrum and 2 from the body; 1LA, one piece from the lesser curvature of the antrum; 1LB, one piece from the lesser curvature of the body; 1GA, one piece from the great curvature of the antrum;1GB, one piece from the great curvature of the body; 1AgB, one piece from the body angularis; 2U, two pieces from the ulcer margin.

In 2009, Shin et al. modified *H. pylori* diagnostic methods and set a validated gold standard according to combination tests results [8]. Since the combination method is the preferred approach in patients with gastritis with atrophy, the defined gold standard may best correlate with active *H. pylori* infection. Our study follows this approach to determine the best biopsy site with highest sensitivity against a background of gastritis with atrophy.

The present study has several findings. First, making a biopsy at the antrum increases the risk of obtaining atrophic gastric mucosa, which is associated higher frequency of false negative results. Second, when body gastritis with atrophy develops, antrum atrophy exists already. Third, antrum biopsy alone has decreased sensitivity when there is moderate-to-severe antrum gastritis with atrophy (CLOtest up to 16% and histology up to 12%) and moderate-to-severe body gastritis with atrophy (CLOtest up to 16.67% and histology up to 8.34%).

First, the frequency of antrum gastritis with atrophy is higher than that of the body. This is consistent with previous studies [22,29]. It is believed that gastritis with atrophy progresses from the antrum initially and extends to the body in the advanced stage [12,17,18]. Thus, the prevalence rate of antrum gastritis with atrophy is significantly higher than that of the body. As such, taking a biopsy at the antrum may yield higher chances of atrophic gastric mucosa than taking one from the body.

Regardless of biopsy site, if the degree of gastritis with atrophy in each specimen is considered, sensitivity decreases as the severity of gastritis with atrophy progresses (Figure 3). This is similar to the findings of Kim et al. [22]. These false negative subjects could be proven to be *H. pylori* infected after combining with other diagnostic methods. Such phenomenon is explained by the increasing rate of sampling error as gastritis with atrophy progresses [29]. Thus, biopsy at antrum increases the risk of getting atrophic gastric mucosa that is associated with higher frequency of false negatives.

Second, applying the aforementioned conclusion on biopsy site and on the degree of gastritis with atrophy, it is interesting to find an opposite results between antrum atrophy and body atrophy. Theoretically, biopsy site at the atrophic area yields poorer sensitivity so single antrum biopsy done in antrum atrophy subjects results in less sensitivity. However, this principle does not apply in single body biopsy performed in body atrophy cases. Instead, the present study reveals a general rule that antrum biopsy results in lower sensitivity in either antrum or body atrophy. This is because when body gastritis with atrophy occurs, antrum atrophy exists already [17,18,29]. In fact, most of the patients here with moderate-to-severe body atrophy (n=23) also have moderate-to-severe antrum atrophy (20/23).

Third, single body biopsy discloses an increased sensitivity as compared with single antrum biopsy in a background of moderate-to-severe antrum gastritis with atrophy (CLOtest up to 16% and histology up to 12%). Similar finding is founded when corpus biopsy is taken in moderate-to-severe body gastritis with atrophy (with a increased sensitivity in CLOtest up to 16.67% and histology up to 8.34%). In contrast, single antrum and single corpus has comparable sensitivity when gastritis with atrophy is normal-to-mild (CLOtest 97.47–95.66 and histology 96.74–98.73). Poor colonization of *H. pylori* in the atrophic mucosa and intestinal metaplasia have been well discussed [9,12]. When performing biopsy-based test in a background of gastritis with atrophy, sampling errors, insufficient bacterial load [9], bacterial migration, bacterial clearance [12], bacterial patchy distribution, and poor mucosal colonization in areas of intestinal metaplasia are common causes of false negative results. Furthermore, as the gastritis with atrophy progresses, the prevalence of *H. pylori* infection decreases. Single specimen sampling at the antrum may miss this relative small but risky patients. And this approach may miss those who could get benefit from eradication. Therefore, the present study recommends additional corpus biopsy to avoid false negative results in moderate-to-severe gastritis with atrophy.

This study has several potential limitations. First, it was conducted in a *H. pylori* endemic area, such that the interpretation and application of the study results should consider the local *H. pylori* prevalence rate. Second, in evaluating gastritis with atrophy by histology, the sampling method used was based on the modified Updated Sydney Classification. The latest approach method to evaluate gastritis with atrophy is OLGA staging, which is composed of multiple antrum and body biopsy specimens [30]. The average grade of antrum and body gastritis with atrophy in these specimens was evaluated while staging was based on the degree and extent of gastritis with atrophy. In this study, only one piece at the antrum and one at the body was used to represent the whole antrum and body mucosal condition. This may be inadequate. However, compared to histology, non-invasive methods like Pepsinogen I/II ratio also disclosed lower values in moderate-to-severe gastritis with atrophy (mean Pepsinogen I/II ratio, 1.96 in moderate-to-severe antrum gastritis with atrophy and 1.79 in moderate-to-severe body gastritis with atrophy). This may eliminate the concern of inadequate sampling in the present study. Third, though we used visual analogue scale to score the gastritis with atrophy, grading of antrum atrophy is most difficult for pathologists since kappa values are very low and this wound be our limitation [10].

## Conclusion

Biopsy-based test should include corpus specimen in cases of moderate-to-severe gastritis with atrophy to avoid false negative results.

## Abbreviations

H. pylori: Helicobacter pylori.

## Competing interests

The authors have no potential conflicts (financial, professional, or personal).

## Authors’ contributions

HC Lin, TS Chen and HC Lan designed the project. TS Chen, FY Chang and AFY Lee performed the experiments. HC Lan wrote the manuscript. TS Chen and HC Lan contributed to the discussion of the data and the revision of the manuscript. All readers read and approved the final manuscript.

## Authors’ information

Writing Assistance: Gene Alzona Nisperos, MD.

## Pre-publication history

The pre-publication history for this paper can be accessed here:

http://www.biomedcentral.com/1471-230X/12/182/prepub
